# How to Minimize the Impact of Pandemic Events: Lessons From the COVID-19 Crisis

**DOI:** 10.34172/ijhpm.2020.115

**Published:** 2020-07-05

**Authors:** Lorenzo Bigiani, Stefano Bigiani, Albertino Bigiani

**Affiliations:** ^1^Dipartimento di Scienze Chimiche, Università di Padova, Padova, Italy.; ^2^Independent Researcher.; ^3^Dipartimento di Scienze Biomediche, Metaboliche e Neuroscienze, Università di Modena e Reggio Emilia, Modena, Italy

**Keywords:** COVID-19, Pandemic, Death Rate, Hospital Stress, Hospital Beds, Health Management

## Abstract

Severe acute respiratory syndrome coronavirus 2 (SARS-CoV-2) is responsible for the current pandemic of coronavirus disease 2019 (COVID-19). This pandemic is characterized by a high variability in death rate (defined as the ratio between the number of deaths and the total number of infected people) across world countries. Several possible explanations have been proposed, but it is not clear whether this variability is due to a single predominant factor or instead to multiple causes. Here we addressed this issue using multivariable regression analysis to test the impact of the following factors: the hospital stress (defined as the ratio between the number of infected cases and the total number of hospital beds), the population median age, and the quality of the National Health System (NHS). For this analysis, we chose countries of the world with over 3000 infected cases as of April 1, 2020. Hospital stress was found to be the crucial factor in explaining the variability of death rate, while the others had negligible relevance. Different procedures for quantifying cases of infection and death for COVID-19 could affect the variability in death rate across countries. We therefore applied the same statistical approach to Italy, which is divided into 20 Regions that share the same protocol for counting the outcomes of this pandemic. Correlation between hospital stress and death rate was even stronger than that observed for countries of the world. Based on our findings and the historical trend for the availability of hospital beds, we propose guidelines for policy-makers to properly manage future pandemics.

## Background

Throughout human history, many pandemics, including cholera, bubonic plague, smallpox, and flu, have caused the death of approximately 300-500 million people worldwide.^1^ Unlike past events, human mobility in today’s hyper-globalized world increases the likelihood of rapid spread of pathogens. In fact, 6 of the 10 pandemic outbreaks of the last century have occurred in the past two decades.^2,3^ During a pandemic, health authorities need to manage the social and economic crisis, as well as reduce the severity of the disease through patient care and treatment. To cope with future pandemic events, and with the aim of saving people’s life, it is important to determine which factors and to what extent they affect the death rate (defined as the ratio between the number of deaths and the total number of infected people) in different countries. This would allow developing a long-term strategy, which in turn would provide the appropriate tools against future plagues.

As reported by the World Bank, poor health outcomes depend on multiple factors, including nature/severity of disease, genetic predisposition, and structural elements.^4^ Policy-makers can only act on structural elements, such as accessibility to hospitals, infrastructures, higher education, and quality of National Health System (NHS). In this context, studies suggest that improving the quality of healthcare may provide the greatest impact in terms of health outcomes, while some structural indexes, particularly the number of hospital beds, seem to be inversely related to health benefits.^4,5^ On the other hand, overwhelmed health systems and other factors (eg, low availability of hospital beds, reduced supplies of disinfectants and ventilators) during pandemics can contribute to a 2.3-fold increase in all-cause mortality.^3^ Nevertheless, European Institutions, such as the European Commission, have encouraged cuts in healthcare and the reduction of hospital beds.^6,7^

Regardless of adopted policy, it is of utmost importance that politicians prepare the country in advance, so that they have all the tools to deal with the pandemic and can proceed promptly in an emergency. This is the only way to avoid panic among people and better manage the crisis, minimizing health, economic, social and political costs.^3^

Since the onset of coronavirus disease 2019 (COVID-19), a disease caused by SARS-CoV-2 (severe acute respiratory syndrome coronavirus 2),^8^ and as of April 1, 2020, 206 countries have suffered from 885 344 cases and 44 214 deaths,^9^ with an average death rate of 4.99%. However, countries have shown variable capacities in the fight against the pandemic, with death rates ranging from less than 0.50% in Australia and Israel to over 11.00% in Italy. The Italian “Istituto Superiore di Sanità” (the national institute for public health) claims^10^ that the high death rate is due to the higher median age of the Italian population (45.5 years)^11^ compared to those in other countries. However, countries like Germany and Japan, with population median age higher than the Italian one (47.1 and 47.3 years, respectively),^11^ show a lower death rate (1.10% and 2.62%, respectively). According to various studies, the quality of NHS should be decisive in determining health benefits against pandemics.^12^ A study published by *The Lancet* indicates that the Italian NHS is better than those in Germany and Japan.^13^ So the issue remains: is there a dominant factor that explains the wide range of death rates among countries since the outbreak of COVID-19 pandemic?

To solve this issue, we correlated the death rate with three possible factors: the hospital stress (defined as the ratio between the number of infected cases and the total number of hospital beds), the population median age, and NHS quality. First, we analysed 25 countries around the world with over 3000 infected people. Then, we repeated the analysis on a single country (Italy) to exclude possible artefacts due to different procedures for the quantification of cases of infection and death among countries.^14^

## Methods

### Data Sources

For world countries (Table S1), data sources were as follows: Coronavirus-Worldometer web site for infected people and death cases at the date of April 1, 2020^9^; World Population Review for population median age^11^; The World Bank for inhabitants^15^; The World Bank and Organization for Economic Co-operation and Development for hospital beds per 1000 inhabitants (HB1000)^16,17^; and *The Lancet* for NHS quality.^13^

For Italy (Table S2), data on infected people and death cases at the date of April 1, 2020, population median age, Regional Health System (RHS) quality, total hospital beds, and inhabitants were from Lab24 web site (the web page of the newspaper “Il Sole 24ore” specifically dedicated to COVID-19 data),^18^ AdminStat,^19^ Quotidianosanità,^20^ Ministero della Salute (Ministry of Health),^21^ and ISTAT (Italian National Statistics Institute),^22^ respectively.

### Sample Selection

Countries in the world that show over 3000 infected cases (N = 25; Table S1) were considered in this study. We adopted this threshold to have a statistically significant data set and to avoid that a reduced number of infected cases could hide the relationship among variables.

Protocols for counting infected cases are not homogeneous across countries.^14^ This can affect values for hospital stress and death rate, thus misleading interpretation of the data. Consequently, we also carried out our analysis on the 20 Italian Regions (Table S2) used as model to verify whether the results observed on a global scale were confirmed under uniform protocol conditions for data acquisition since, in Italy, protocols for the quantification of cases of infection and death are defined by the government.^23,24^

### Statistical Analysis

Statistical analysis was performed using SPSS 25.0 software (version 25.0. IBM Corp., Armonk, NY, USA). To verify correlations among variables, we used multivariable regression analysis fixing death rate as a dependent variable and all the other factors as independent variables (hospital stress, population median age, and NHS or RHS quality), both for the selected countries of the world and for the Italian Regions. This test was particularly suitable for studying the correlation between two or more independent variables with a dependent variable^25^ and for establishing the contribution of each of them to a single event, thus excluding the effect of the others based on the partial correlation coefficients (r) and *P* values.^26-28^

## Results

### MODEL-1: World Countries

**Figure 1:**
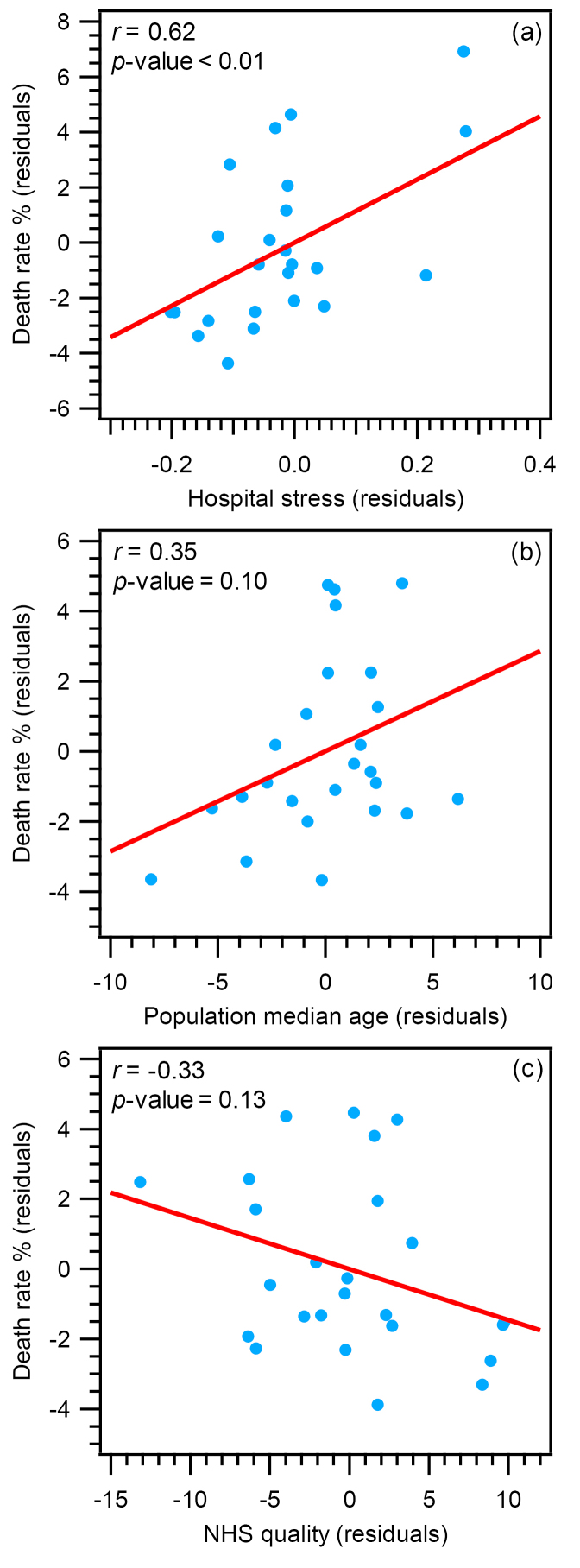
**MODEL-1.** Partial regression plots between death rate and (a) hospital stress, (b) population median age, (c) and NHS quality, for the selected 25 countries of the world. Partial correlation coefficients (*r*) and *P* values are reported as insets. Abbreviation: NHS, National Health System.

First, we tested the model for the 25 selected countries of the world (MODEL-1). Multivariable regression analysis (Table S3) indicated at least one of the independent variables was significantly related to the death rate (*R* = 0.66, *F* = 5.28, *P* value < .01). Figure 1 shows the scatterplots of the partial correlation for all variables in MODEL-1. Hospital stress was strongly and positively related to death rate (*r* = 0.62, *β* = 0.62, *P* value < .01; Figure 1a). On the contrary, the population median age (Figure 1b), and the quality of NHS (Figure 1c) were not significantly related to death rate: *r* (absolute value) < 0.35, *β* (absolute value) < 0.43, and *P* value > .05 (see Table S3 for details). For each variable, the univariate regression plot is reported in Figure S1.

### MODEL-2: Italian Regions

We then applied the same approach to study the specific case of Italy (MODEL-2). As described above, this country is organized into 20 regions that share the same protocols for data acquisition. Furthermore, the Italian Regions show variability in death rate (3.10%-16.66%), population median age (42.15-48.46 years), RHS quality (91.60-107.50), and hospital stress (0.10-1.28), reflecting the situation observed across countries around the world. The results of multivariable regression analysis (Table S4) indicated that even in the case of Italy at least one of the independent variables was significantly related to the death rate (*R* = 0.77, *F* = 7.92, *P* value < .01).

**Figure 2:**
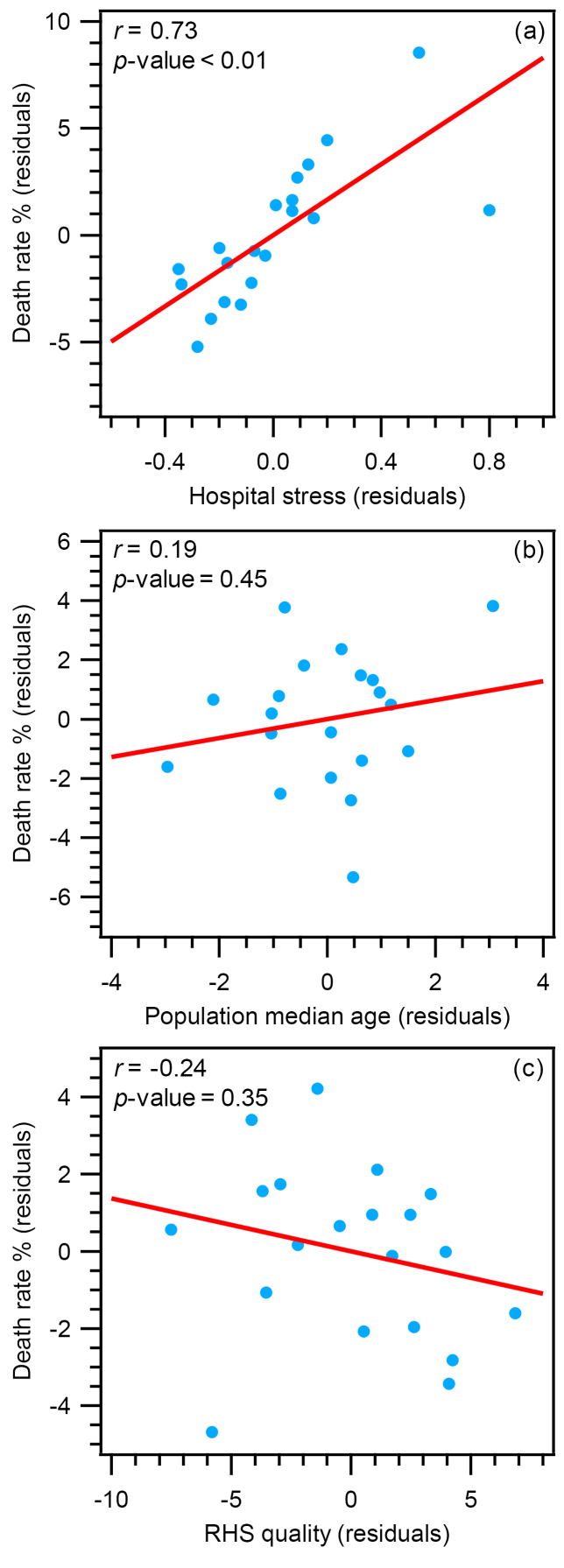
**MODEL-2.** Partial regression plots between death rate and (a) hospital stress, (b) population median age, (c) and RHS quality, for the 20 Italian Regions. Partial correlation coefficients (*r*) and *P* values are reported as insets. Abbreviation: RHS, Regional Health System.

Figure 2 shows the scatterplots of the partial correlations for all variables in MODEL-2. Consistent with MODEL-1, the partial correlation between hospital stress and death rate (Figure 2a) was also significant (*r* = 0.73, *β* = 0.85, *P* value < .01). Furthermore, partial correlations between the population median age (Figure 2b), or the RHS quality (Figure 2c) and the death rate were not statistically significant, with *r* (absolute value) < 0.24, *β* (absolute value) < 0.21, and *P* value > .05 (see Table S4). For each variable, univariate regression plot is reported in Figure S2.

These results confirmed the strength of the correlation between hospital stress and death rate also excluding possible artefacts due to different methodology in counting cases of infection and death.

Two outlying observations occurred in the Italia data (Lombardia and Valle d’Aosta with hospital stress >1; Table S2). We therefore tested the possible effect of these outliers in driving the regression analysis (Figure S3 and Table S5). Without the outliers, the correlation between death rate and hospital stress became even stronger (*r* = 0.84, *β* = 1.16, *P* value < .01; seeFigure S3a and Table S5). Interestingly, by removing outliers, death rate was related also to the RHS quality (*r* = -0.63, *β* = -0.65, *P* value = .01; see Figure S3c and Table S5). This result suggests that when hospital stress is <1 (non-overcrowded hospitals), RHS quality plays a significant role in determining the death rate. On the other hand, RHS quality becomes less important when hospitals cannot accept and treat all patients (hospital stress >1).

### The Impact of Hospital Stress on the Death Rate

**Figure 3:**
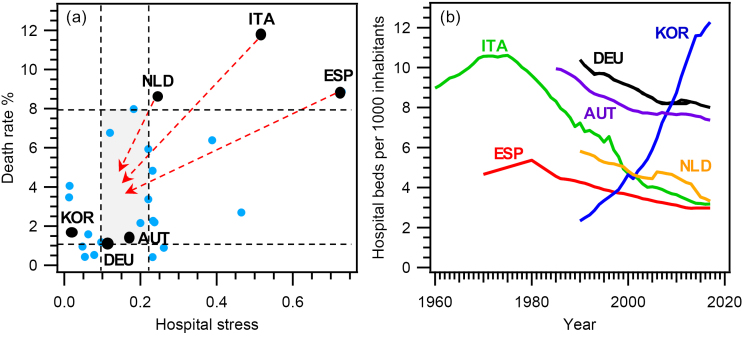
The Impact of Hospital Stress on Death Rate. (a) Scatterplot of hospital stress *vs.* death rate for all countries with more than 3000 COVID-19 infected cases as of April 1, 2020 (N = 25). Black dots indicate representative countries chosen as follows: countries with the highest death rate (ESP, ITA, NLD), and countries with the highest HB1000 (AUT, DEU, KOR). Hospital stress and death rate are reported in Table S1. See text for the meaning of horizontal and vertical dashed lines. (b) Historical trend of hospital beds per 1000 inhabitants (HB1000) for countries marked with black dots in (a). Abbreviations: COVID-19, coronavirus disease 2019; ESP, Spain; ITA, Italy; NLD, the Netherlands; AUT, Austria; DEU, Germany; KOR, South Korea.

Hospital stress is the ratio between the number of infected people and total number of hospital beds: the former depends on multiple factors, including the characteristics of the pathogen, while the latter depends only on political decisions. We therefore performed a further analysis to determine how much these policy choices could affect the death rate. To this end, we assessed the impact of the number of hospital beds per 1000 inhabitants (HB1000) on the death rate. For simplicity, we have considered the countries with the highest death rates and low HB1000s (Italy [ITA], the Netherlands [NLD], Spain [ESP]), and those exhibiting low death rates and the highest values of HB1000 (Austria [AUT], Germany [DEU], South Korea [KOR]) (Figure 3). Our question was: what would happen to the death rate in ESP, ITA, and NLD if these countries had a HB1000 like that of AUT, DEU, and KOR?

To answer this question, we first calculated the hospital stress for ESP, ITA, and NLD using a HB1000 of 9.21, which corresponds to the average value of HB1000 for AUT, DEU, and KOR (Table S1). In this scenario, hospital stress values for ESP, ITA, and NLD would vary from 0.09 (NLD) to 0.24 (ESP), since the Italian value would be 0.19. In Figure 3a, this range is indicated by two vertical dashed lines. The death rate for the hospital stress included between the two vertical lines varies across world countries from 1.10% to 7.98% (horizontal dashed lines in Figure 3a). These values allow identifying a rectangular area (grey colour in Figure 3a), which would include ESP, ITA, and NLD if they had an HB1000 of 9.21 (red arrows in Figure 3a). Using those limits for the death rate, we could estimate its change in ESP, ITA, and NLD for a HB1000 of 9.21: in detail, we would get a 10%-88%, 32%-91%, and 7%-87% drop for ESP, ITA, and NLD, respectively. In other words, in these countries it would have been possible to save from 4900 (lower percentage drop) to 20 300 (higher percentage drop) people during COVID-19 pandemic. This outcome highlights the great potential of specific actions aimed at increasing HB1000, along with a greater number of physicians and nurses to be associated with the increased number of hospital beds, to reduce hospital stress.

HB1000 includes curative, rehabilitative, long-term care and other beds in hospitals and is useful to compare the overall capacity of NHS in different countries.^17^ During pandemics, most if not all the hospital beds are converted and used for infected people,^29^ as in the case of the current COVID-19 pandemic. As a result, HB1000 is a good indicator of the resources available to help hospitalized patients during pandemics.

Figure 3b shows how HB1000 evolved in the 1960–2020 period in the six countries used for the above analysis. Although only ITA’s data set covers the entire period, some useful information can be obtained for all these countries. ITA and ESP show a similar trend with two distinct phases: HB1000 increases until the mid/late 1970s (10.61 and 5.36, respectively) and then it drops to current levels (3.18 and 2.97, respectively) with a reduction of 70% for ITA and 45% for ESP. AUT and DEU have a similar downward trend until 2002 and 2007, respectively. Then, after a stable period until 2012, HB1000 drops to 7.37 and 8.00, respectively. Even so, it remains 2.5 times higher than that of ITA and in ESP. Since 1990, HB1000 has fallen by 43% in NLD (from 5.82 to 3.32), approaching values such as those of ITA and ESP. KOR exhibits peculiar trend, opposite to that described for European countries. Since 1990, HB1000 has increased rapidly from 2.33 to 12.27 (+426%), thus surpassing also DEU and the highest value ever seen in ITA in the 1970s (10.61). These data highlight that the common policy followed by European countries has led to the reduction of hospital beds. Indeed, this policy fits well with the cut-back programs on healthcare suggested by the European Institutions.^6,7^ According to our analysis, this policy is probably a concurrent cause responsible for the high death rates observed in some countries. If ESP, ITA, and NLD had had a HB1000 of 9.21, it would have been possible to save thousands of people (4900-20 300) during COVID-19 pandemic (Figure 3b).

## Discussion

Much effort has been devoted to identifying factors “*needed for health system resiliency to infectious disease outbreaks and natural hazards.”*^12^ However, as far as we know, a clear quantification of the impact of these factors on the death rate is missing.^12^ This information is needed to design specific interventions during pandemics. According to our results, hospital stress has a significant impact on the death rate, whereas population median age, or NHS/RHS quality do not. Therefore, our findings indicate that the hospital stress plays a crucial role in addressing pandemics. Although this is intuitively obvious, it is worth pointing out that national and international authorities seem to underestimate this factor in their analyses.^6,7,10^ On the contrary, our study suggests that the availability of hospital beds for infected people is a limiting factor in the correct management of the pandemic crisis.

Although our results showed that the different death rate across countries was mainly influenced by hospital stress and not by population median age or NHS quality, other variables could also play a role. Several studies have shown that environmental conditions such as temperature,^30-32^ humidity,^31,32^ and latitude,^33^ seem to be related to the spread of viruses. However, when these variables are included, together with other factors, in a multivariable model, these correlations are weaker or no longer significant.^34^ In this context, public health interventions (restrictions of people gatherings, social distancing, or closure of non-essential activities) seem to become the key variables.^34^

This observation is consistent with data from the Italian Regions, where the death rate in the North is twice as high as in the Center-South (13.03% and 6.65%, respectively; Figure S4) during the current COVID-19 pandemic. It is worth noting that the death rates induced by similar diseases, such as flu and pneumonia, have shown no appreciable difference in the past years between the North and the Center-South.^35,36^ Thus, the variation in the death rate across Italian Regions during COVID-19 pandemic is not due to pre-existing causes. On the other hand, when the Italian government established the lockdown at national level (March 11, 2020) the total number of infected people in the North was almost 8 times greater than that assessed in the Center-South (see Table S2). This specific intervention likely limited the spread of the virus from the North to the Center-South, leading to a lower hospital stress in the latter (0.81 and 0.20, respectively) and consequently to a lower death rate.

Overall, our results suggest that all countries have the potential to significantly improve their readiness and resilience against pandemics by increasing the total number of hospital beds.

### “You must know the past to understand the present and orient the future” (Thucydides, 5th century BC)

Based on our findings, we would like to propose the following suggestions for the policy-makers:

1. Countries should follow South Korea’s example by increasing funding for the construction of new hospitals and related infrastructure, thus reversing the current trend observed in almost all countries analysed. This action would increase the overall capacity of NHS, which in turn would allow a better care for infected people. A stronger NHS would provide more time for proper crisis management before pandemic gets out of control. In fact, without efficient accessibility to hospital care, the benefits of other actions, such as decision-making/coordination (school closure, quarantine, antiviral therapy, social distancing, contact tracing), could be less effective.
2. Since it is impossible to predict the infectivity and lethality of future pandemics, it is necessary to prepare an emergency plan that include the ability to quickly build temporary hospitals to properly manage the peak of infection. This would rapidly increase the responsiveness of the NHS.

## Conclusion

Since the outbreak of COVID-19 pandemic, several hypotheses have been proposed to explain the great variability in the death rate across countries. Our study provides evidence that one of the most crucial factors affecting the death rate is the availability of hospital beds (as indicated by the hospital stress). With the aim of reducing deaths during the pandemic, our results suggest that countries should implement policies to increase hospital beds in order to avoid the saturation of NHS.

## Acknowledgements

We thank Alessandro Bigiani, Davide Bigiani, and Silva Gavioli for helpful discussion and suggestions.

## Ethical issues

Ethical approval was not required as the study was based on secondary data.

## Competing interests

Authors declare that they have no competing interests.

## Authors’ contributions

LB designed this study. LB and SB performed statistical analysis. LB, SB, and AB discussed the data and wrote the paper.

## Authors’ affiliations

^1^Dipartimento di Scienze Chimiche, Università di Padova, Padova, Italy. ^2^Independent Researcher. ^3^Dipartimento di Scienze Biomediche, Metaboliche e Neuroscienze, Università di Modena e Reggio Emilia, Modena, Italy.

## Supplementary file


Supplementary file 1 contains Tables S1-S5 and Figures S1-S4.
