# Supplementary material for: How to Minimize the Impact of Pandemic Events: Lessons From the COVID-19 Crisis
Source: Int J Health Policy Manag. 2020 Jul 5;9(11):469–74. doi: 10.34172/ijhpm.2020.115 (PMC7719203; doi:10.34172/ijhpm.2020.115)
Supplement: Supplementary file 1 — contains Tables S1-S5 and Figures S1-S4. [file ijhpm-9-469-s001.pdf]

Supplementary file 1

Table S1. Data for countries in the world with over 3000 infected cases as of April 1, 2020

Country	Total infects[1]	Total deaths[1]	Median age (year)[2]	NHS quality index[3]	Population (millions)[4]	Hospital beds per 1000 inhabitants (HB1000)[5,6]	Death rate (%)*	Hospital stress*
Australia	4,864	21	38.70	96	23.49	3.84	0.43	0.05
Austria	10,501	146	44.00	94	8.47	7.37	1.39	0.17
Belgium	13,964	828	41.40	93	11.23	5.64	5.93	0.22
Brazil	5,861	203	32.00	64	201.47	2.30	3.46	0.01
Canada	8,612	101	42.20	94	35.54	2.50	1.17	0.10
Chile	3,031	16	34.40	78	17.82	2.17	0.53	0.08
China	81,554	3,312	37.40	78	1392.73	4.20	4.06	0.01
Czech Republic	3,33	32	42.10	89	10.52	6.63	0.96	0.05
Denmark	3,092	104	42.20	92	5.61	2.50	3.36	0.22
France	52,128	3,523	41.40	92	66.99	6.50	6.76	0.12
Germany	74,508	821	47.10	92	82.93	8.30	1.10	0.11

Iran	47,593	3,036	30.30	72	81.80	1.50	6.38	0.39
Ireland	3,235	71	36.80	95	4.61	2.96	2.19	0.24
Israel	5,591	23	29.90	85	8.06	2.99	0.41	0.23
Italy	105,792	12,428	45.50	95	60.43	3.40	11.75	0.51
Netherlands	13,614	1,173	42.60	96	16.80	3.32	8.62	0.24
Norway	4,828	43	39.20	97	5.14	3.60	0.89	0.26
Portugal	8,251	187	42.20	86	10.46	3.39	2.27	0.23
South Korea	9,887	165	41.80	90	50.42	12.27	1.67	0.02
Spain	102,136	9,053	42.70	92	46.72	3.00	8.86	0.73
Sweden	4,947	239	41.20	95	9.61	2.22	4.83	0.23
Switzerland	17,137	461	42.20	96	8.14	4.53	2.69	0.46
Turkey	13,531	214	30.90	74	76.90	2.81	1.58	0.06
UK	29,474	2,352	40.50	90	63.65	2.54	7.98	0.18
USA	189,711	4,099	38.10	89	327.17	2.90	2.16	0.20

* Death rate % is the ratio between the number of deaths and the total number of infected people multiplied by 100. Hospital stress is the ratio between the number of infected people and the number of hospital beds. This latter is obtained by multiplying hospital beds per 1000 inhabitants (HB1000) by population.

NHS = National Health System

Table S2. Data for the Italian Regions

Region	Total infects (April 1, 2020)[7]	Total infects (March 11, 2020)[7]	Total deaths (April 1, 2020)[7]	Median age (year)[8]	RHS quality index[9]	Population (millions)[10]	Hospital beds[11]	Death rate (%)	Hospital stress
Abruzzo	1,401	38	115	45.67	96.40	1.31	4,415	8.21	0.32
Basilicata	226	8	7	45.31	98.70	0.56	1,862	3.10	0.12
Calabria	659	19	36	43.98	89.10	1.95	5,796	5.46	0.11
Campania	2,092	154	133	42.15	91.60	5.80	18,003	6.36	0.12
Emila-Romagna	14,074	1739	1,644	45.70	107.30	4.46	17,395	11.68	0.81
Friuli Venezia Giula	1,593	126	113	47.00	103.70	1.22	4,357	7.09	0.37
Lazio	3,095	150	162	44.58	101.50	5.88	20,818	5.23	0.15
Liguria	3,416	194	428	48.46	101.30	1.55	5,715	12.53	0.60
Lombardia	43,208	7280	7,199	44.74	104.10	10.06	37,795	16.66	1.14
Marche	3,825	479	452	46.09	104.90	1.53	5,231	11.82	0.73
Molise	144	16	9	46.28	101.70	0.31	1,228	6.25	0.12
Piemonte	9,301	501	854	46.54	102.50	4.36	16,638	9.18	0.56

Puglia	1,803	77	110	44.23	98.00	4.03	12,543	6.10	0.14
Sardegna	722	37	31	46.33	95.80	1.64	5,79	4.29	0.12
Sicilia	1,647	83	81	43.53	93.80	5.00	15,825	4.92	0.10
Toscana	4,608	320	244	46.52	104.20	3.73	12,025	5.30	0.38
Trentino Alto Adige	3,117	152	240	43.22	107.50	1.07	4,198	7.70	0.74
Umbria	1,078	46	37	46.49	105.20	0.88	3,271	3.43	0.33
Valle d'Aosta	628	20	56	45.63	101.80	0.13	489	8.92	1.28
Veneto	9,155	1023	477	45.10	105.30	4.91	17,513	5.21	0.52

* Death rate % is the ratio between the number of deaths and the total number of infected people multiplied by 100. Hospital stress is the ratio between the number of infected people and the number hospital beds.
RHS = Regional Health System

Table S3. Multivariable regression analysis for the model of the 25 selected countries and with death rate as dependent variable

**MODEL-1: World countries**			
**Complete model**	***F***	***P* Value**	***R***
all independent variables	5.28	<0.01	0.66
**Partial correlation**	***β***	***P* Value**	**Partial *r***
hospital stress	0.62	<0.01	0.62
population median age	0.43	0.10	0.35
NHS quality	-0.40	0.13	-0.33

Table S4. Multivariable regression analysis for the model of the Italian Regions and with death rate as dependent variable

**MODEL-2: Italian Regions**			
**Complete model**	***F***	***P* Value**	***R***
all independent variables	7.92	<0.01	0.77
**Partial correlation**	***β***	***P* Value**	**Partial *r***
hospital stress	0.85	<0.01	0.73
population median age	0.14	0.45	0.19
RHS quality	-0.21	0.35	-0.24

Table S5. Multivariable regression analysis for the model of the Italian Regions without the two outliers and with the death rate as dependent variable

**MODEL-2: Italian Regions**			
**Complete model**	***F***	***P* Value**	***R***
all independent variables	13.90	<0.01	0.87
**Partial correlation**	***β***	***P* Value**	**Partial *r***
hospital stress	1.16	<0.01	0.84
population median age	0.26	0.10	0.42
RHS quality	-0.65	0.01	-0.63

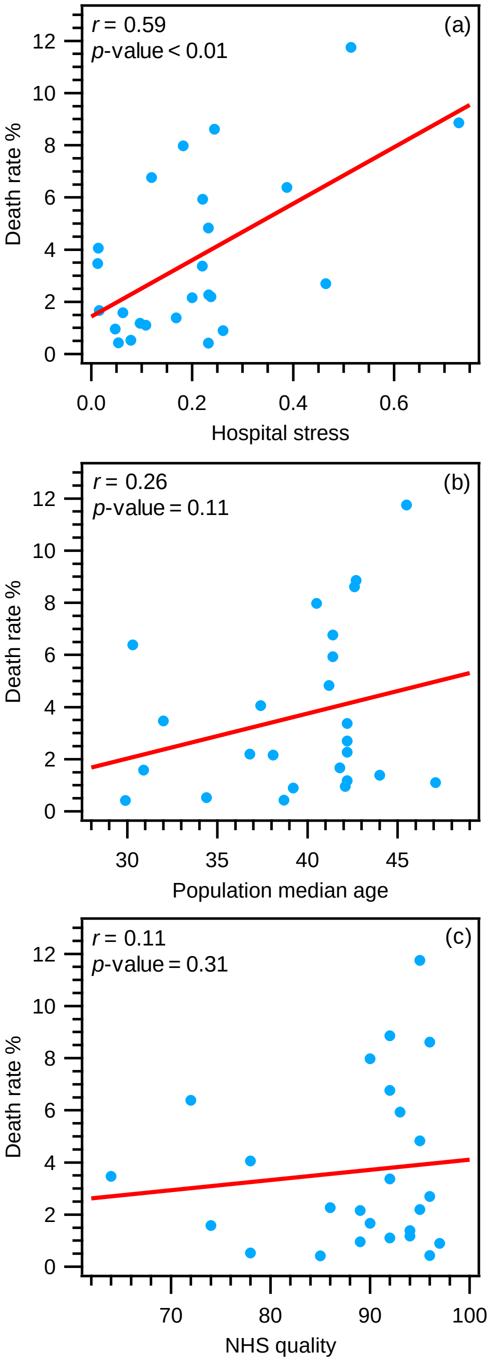

Figure S1. MODEL-1. Scatterplots for the univariate correlations between the death rate and (a) hospital stress, (b) population median age, (c) and NHS quality, for the 25 selected countries of the world. Pearson correlation coefficients (*r*) and *P* values are reported as insets.

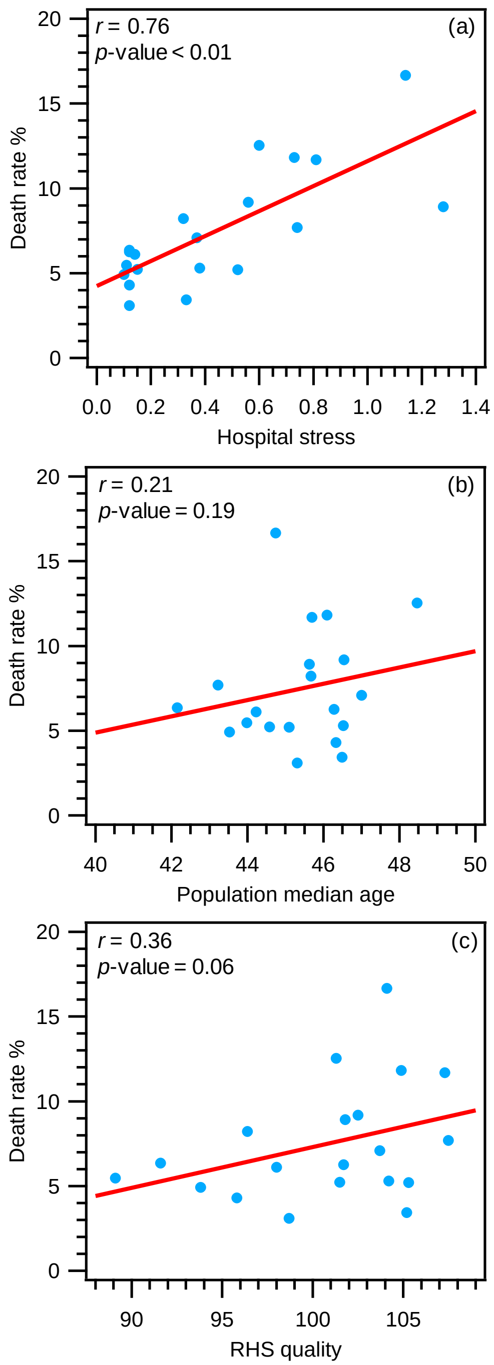

Figure S2. MODEL-2. Scatterplots for the univariate correlations between the death rate and (a) hospital stress, (b) population median age, (c) and RHS quality, for the Italian Regions. Pearson correlation coefficients (*r*) and *P* values are reported as insets.

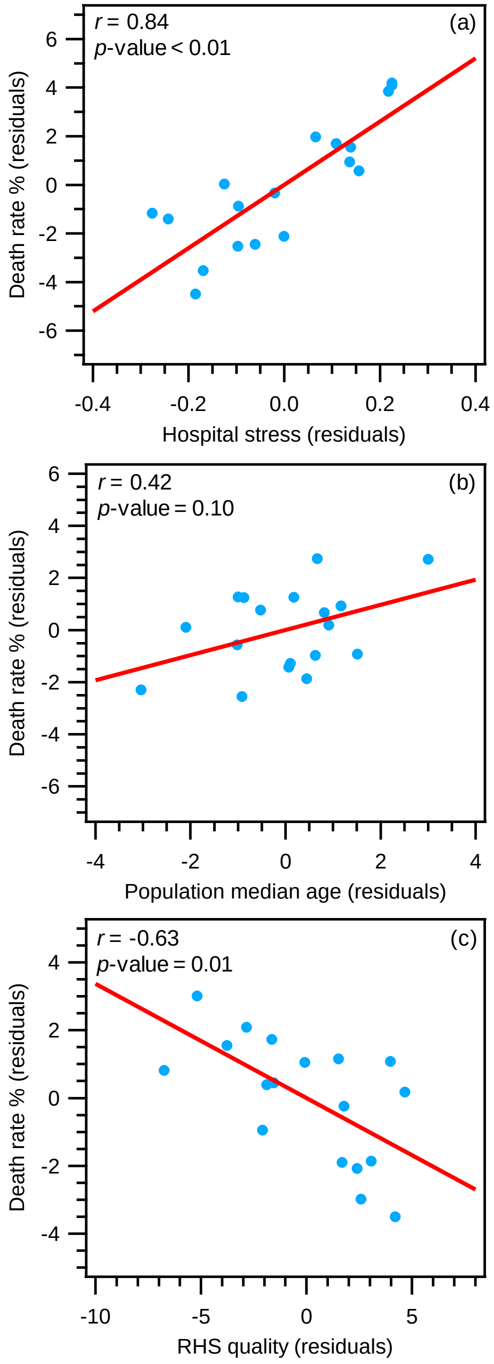

Figure S3. **MODEL-2.** Partial regression plots between the death rate and (a) hospital stress, (b) population median age, (c) and RHS quality, for the Italian Regions without the two outliers. Partial correlation coefficients (*r*) and *P* values are reported as insets.

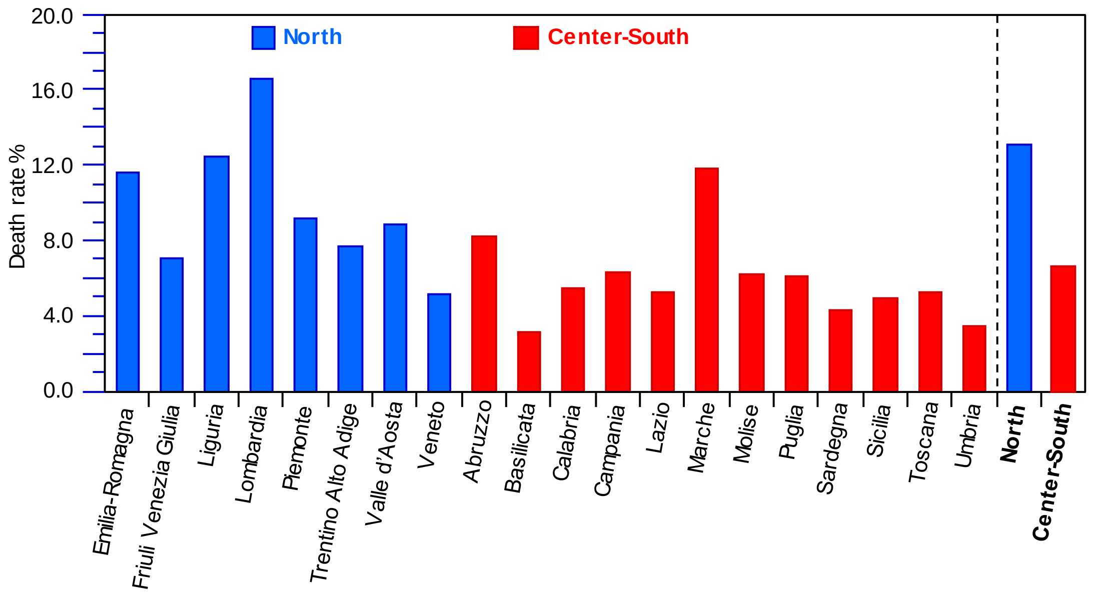

Figure S4. Death rate during COVID-19 pandemic for all the Italian Regions (see Table S2) and for the two macro areas, North and Center-South. Death rate for these macro areas is calculated as the ratio between the total number of deaths and the number of infected people in the regions included in each macro area.

References

1. COVID-19 CORONAVIRUS PANDEMIC. Coronavirus-Worldometer Web site: https://www.worldometers.info/coronavirus/. Updated April 1, 2020. Accessed April 1, 2020.
2. Countries by median Age 2018. World Population Review Web site: https://worldpopulationreview.com/countries/median-age/. Updated 2020. Accessed April 1, 2020.
3. GBD 2016 Healthcare Access and Quality Collaborators. Measuring performance on the Healthcare Access and Quality Index for 195 countries and territories and selected subnational locations: a systematic analysis from the Global Burden of Disease. Study 2016. *Lancet* 2018; 391(10136): 2236-2271. doi: 10.1016/S0140-6736(18)30994-2.
4. Population, total. The World Bank Web site: https://data.worldbank.org/indicator/sp.pop.totl. Updated 2019. Accessed April 1, 2020.
5. Hospital beds (per 1,000 people). The World Bank Web site: https://data.worldbank.org/indicator/sh.med.beds.zs. Updated 2019. Accessed April 1, 2020.

6. Hospital beds (indicator). OECD Data Web site: https://data.oecd.org/healtheqt/hospital-beds.htm. Updated 2020. Accessed April 1, 2020.
7. Coronavirus in Italia, i dati e la mappa. Lab24 Web site: https://lab24.ilsole24ore.com/coronavirus/. Updated April 1, 2020. Accessed April 1, 2020.
8. Mappe tematiche, curiosità, confronti e classifiche per i comuni, le province e le regioni sulla base di 20 indicatori socio-demografici. AdminStat Web site: https://ugeo.urbistat.com/AdminStat/it/it/classifiche/eta-media/regioni/italia/380/1. Updated January 1, 2020. Accessed April 1, 2020.
9. La performance sanitaria. Indice di misurazione e valutazione dei sistemi regionali italiani. Quotidianosanità Web site: https://www.quotidianosanita.it/allegati/allegato8783063.pdf. Updated June 6, 2019. Accessed April 1, 2020.
10. Popolazione residente al 1° Gennaio. Istat Web site: http://dati.istat.it/Index.aspx?DataSetCode=DCIS_POPRES1. Updated January 1, 2020. Accessed April 1, 2020.
11. Posti letto per struttura ospedaliera. Ministero della Salute Web site: http://www.dati.salute.gov.it/dati/dettaglioDataset.jsp?menu=dati&idPag=18. Updated December 31, 2019. Accessed April 1, 2020.
